# Bacterial pericardial effusion secondary to endobronchial ultrasound guided needle aspiration

**DOI:** 10.1002/rcr2.1290

**Published:** 2024-02-05

**Authors:** Benjamin Salwen, Dan Frechtling, Wadih Chahine, Jaime Palomino

**Affiliations:** ^1^ Department of Medicine Tulane University School of Medicine New Orleans Louisiana USA; ^2^ Department of Radiology Tulane University School of Medicine New Orleans Louisiana USA; ^3^ Division of Pulmonary, Critical Care and Environmental Medicine Tulane University School of Medicine New Orleans Louisiana USA; ^4^ Department of Pulmonary and Critical Care Medicine, Southeast Louisiana Veterans Healthcare System New Orleans Louisiana USA

**Keywords:** bronchoscopy, EBUS‐TBNA, pericardial effusion

## Abstract

Endobronchial ultrasound‐guided transbronchial needle aspiration (EBUS‐TBNA) is a widely used procedure in lung cancer diagnosis with few serious complications. We present a rare case of pericardial effusion secondary to EBUS‐TBNA. An 80‐year‐old male with interstitial lung disease, developed a pericardial effusion composed exclusively of oropharyngeal flora following EBUS‐TBNA. Bacterial pericardial effusion following EBUS‐TBNA has only been reported in the literature seven previous times. The majority of these cases reported a biopsy of the 4R lymph node. This case highlights the potential risk of pericardial effusion when sampling lymph nodes, particularly station 4R, in patients with a high‐riding superior pericardial recess.

## Graphical abstract

We present a rare case of pericardial effusion secondary to EBUS‐TBNA. This case highlights the potential risk of pericardial effusion when sampling lymph nodes, particularly station 4R, in patients with a high riding superior pericardial recess.

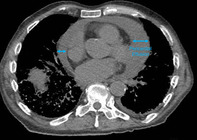

## INTRODUCTION

Endobronchial ultrasound‐guided transbronchial needle aspiration (EBUS‐TBNA) is widely used for the diagnosis and staging of lung cancer. It is well tolerated with few reported complications. Due to its low complication rate and high diagnostic yield it is widely used for examining mediastinal and hilar lymph nodes. Per literature review pericardial effusion secondary to EBUS‐TBNA has only been reported 7 times.^1^, ^2^, ^3^, ^4^, ^5^, ^6^, ^7^

## CASE REPORT

An 80‐year‐old male patient with a history of interstitial lung disease, former tobacco use, and diabetes mellitus was admitted for 3 days of non‐productive cough and dyspnea. He received a high‐resolution computed tomography scan (CT) revealing chronic fibrosis, a new mass in the right upper lobe and enlarged mediastinal and right hilar lymph nodes. EBUS‐TBNA was used to biopsy the endobronchial mass and two lymph nodes (4R and 11RS). Cryobiopsy was used for both mass and lymph nodes following needle biopsy due to concern for necrotic material to ensure good diagnostic yield. Biopsy of both mass and lymph nodes were positive for malignant cells. He tolerated the procedure well, with expected but minimal bleeding at the site of the mass following cryobiopsy, which was controlled with cold saline and argon plasma coagulation.

**FIGURE 1:**
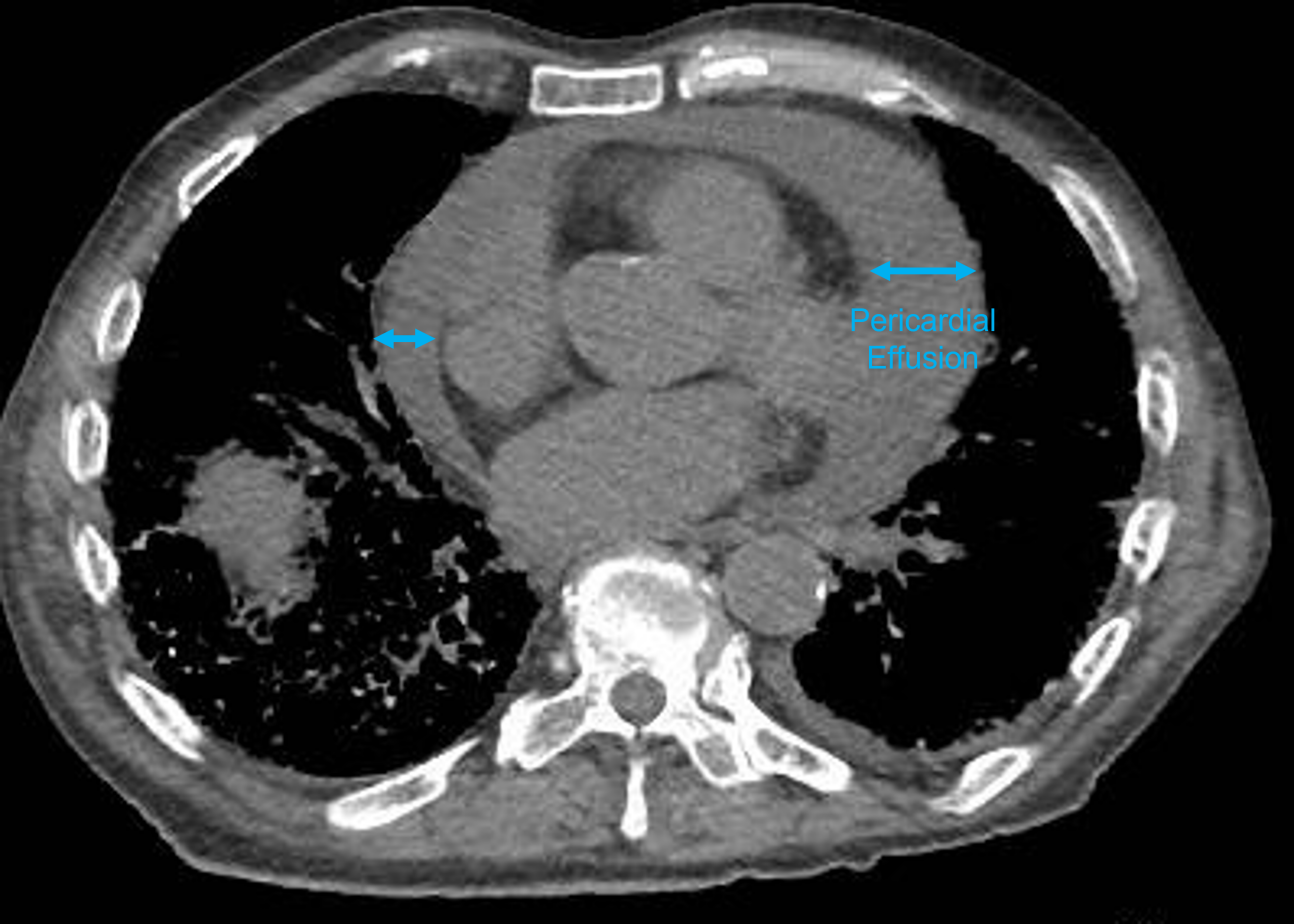
Axial view of computed tomography chest, demonstrating pericardial effusion.

Four days following the procedure, he began to have chest pain, shortness of breath and an increasing oxygen requirement. An EKG performed at the time showed diffuse ST elevations, however, echocardiogram done at this time was unremarkable. Notable labs at the time were as follows: two sets of troponins were negative (0.3 and 0.3), elevated CRP (CRP 16.25) and ESR (ESR 57). Autoimmune workup previously conducted for ILD was negative. Respiratory viral PCR, which included enteroviruses such as Coxsackie virus was also negative. He was initiated on high‐dose aspirin and colchicine for suspected pericarditis of unknown cause. One week following EBUS, he was noted to have rising leukocytosis (15.7–23.6) with neutrophil predominance. Later that day, the patient became hypotensive. A CT abdomen chest pelvis was conducted to determine the potential source of infection. This revealed interval development of a large pericardial effusion (Figure 1) which in conjunction with the onset of hypotension was suggestive of evolving tamponade physiology.

Bedside echocardiogram redemonstrated the effusion and he received an emergent pericardiocentesis, 800 mL of yellow/turbid fluid was drained. Following pericardiocentesis he was empirically started on vancomycin, cefepime, and metronidazole. Pericardial fluid studies showed 125 K WBC with 60% segmented neutrophils, gram stain showed gram positive cocci in pairs and clusters. Cytology of the pericardial fluid showed no evidence of malignant cells and was predominantly inflammatory cells with scattered mesothelial cells. Culture from pericardiocentesis grew *Actinomyces odontolyticus*, *Streptococcus constellatus*, *Eikenella corrodens*, *Aggregatibacter segnis*, and *Veillonella parvula*. There was no growth on peripheral blood culture. His antibiotics were narrowed to ampicillin sulbactam. A decrease in the amount of pericardial fluid was observed on follow‐up TTE. His medical condition improved, and he was discharged home with strict return precautions and plans for palliative oncologic treatment.

## DISCUSSION

**FIGURE 2:**
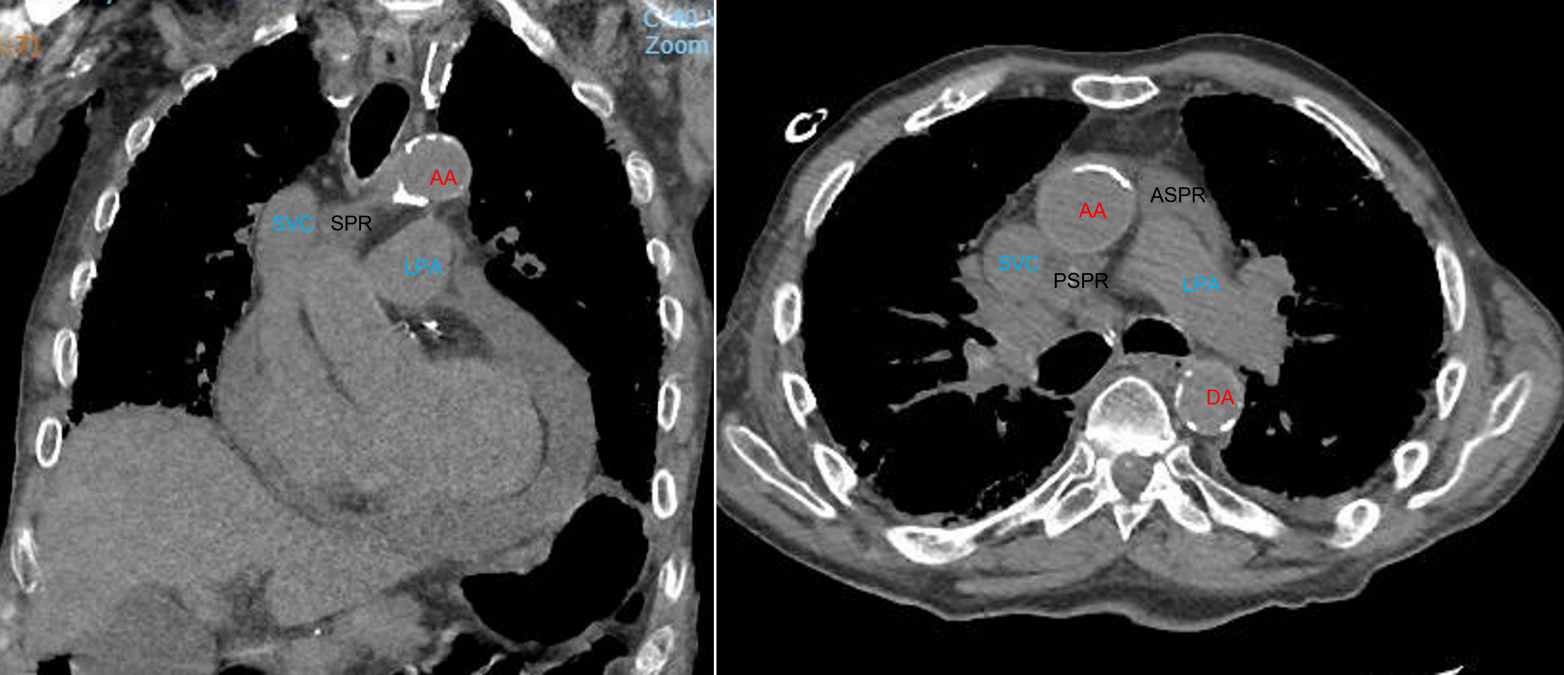
Coronal and axial views of computed tomography chest, demonstrating high riding superior pericardial recess (SPR) both anterior (ASPR) and posterior (PSPR) and its distinction from the ascending aorta (AA), superior vena cava (SVC), left pulmonary artery (LPA) and the descending aorta (DA).

We present a rare case of bacterial pericardial effusion following EBUS‐TBNA. After a biopsy of stations 4R and 11RS, our patient developed a pericardial effusion. Our patient did not have an alternative source for his pericardial effusion, other than direct inoculation from his EBUS‐TBNA. This was further supported by both the time course of his infection and the bacterial species of his pericardial fluid being comprised solely of oropharyngeal bacteria. On review of the literature, 5/7 previous cases included biopsies of 4R.^1^, ^2^, ^3^, ^6^, ^7^ The remaining cases which did not mention 4R, did not specify which lymph nodes were biopsied. Anatomically the biopsied lymph nodes are not normally contiguous with the pericardium, 4R is superior lateral and 11RS is lateral. However, in some individuals the superior pericardial recess can have supra‐aortic extension into the high right paratracheal region,^8^ this appears to be the case in our patient (Figure 2). The prevalence of this anatomic anomaly is not well described in the literature, with a few observational studies reporting its prevalence between 2% and 6%.^9^ We hypothesize that in our case, there was direct seeding of the pericardium with oropharyngeal flora from the EBUS scope as the needle passed through the superior recess to sample 4R.

Bacterial pericardial effusion following EBUS‐TBNA is a rare, but potentially life‐threatening complication. Clinicians should be aware that a high riding superior pericardial recess can mimic paratracheal lymphadenopathy complicating lymph node sampling. Due to the severity of this complication, it is important to be aware of this anatomical variant and the increased possibility that pericardial effusion can occur due to it, especially when sampling 4R lymph nodes.

## AUTHOR CONTRIBUTIONS

**Benjamin Salwen**: Conceived, designed, drafted, and edited the manuscript. In charge of final manuscript with the assistance of the other co‐authors. **Dan Frechtling**: Helped conceive, write, and edit the manuscript. Assisted in drafting the work and reviewing it. **Wadih Chahine**: Consultant radiologist, assisted in selection and review of all imaging. **Jaime Palomino**: Provided important input and review of content. Faculty advisor, gave final approval of the version to be published.

## CONFLICT OF INTEREST STATEMENT

None declared.

## ETHICS STATEMENT

The authors declare that appropriate written informed consent was obtained for the publication of this manuscript and accompanying images.

## Data Availability

The data that support the findings of this study are available on request from the corresponding author. The data are not publicly available due to privacy or ethical restrictions.
